# A New Endoscopic Technique for Examination of Esophageal Stenosis: The Funnel-shaped Transparent Cap Technique

**DOI:** 10.1155/DTE.5.131

**Published:** 1999

**Authors:** O. Katayama, N. Wakabayashi, S. Ishihama, I. Ohi

**Affiliations:** 1 Clinical Laboratory Saitamaken-Saiseikai Kurihashi Hospital Kouemon 714-6 Kurihashi-Cho, Kitakatsushika-gun Saitama 349-1105 Japan; 2 Endoscope Technical Division Toshiba Medical Co. Tokyo 113-8456 Japan; 3 Laboratory Tokyo Women's Medical College Daini Hospital Tokyo 116-0011 Japan

## Abstract

We have devised a funnel-shaped transparent cap for the endoscopic diagnosis and treatment of stenosis in the digestive tract. This funnel-shaped cap is made of highly transparent methacrylic resin. A 73-year-old woman with reflux esophagitis (categorized as grade D by the Los Angeles Classification) visited our hospital with the chief complaint of dysphagia. She was examined using an endoscope equipped with a transparent vinyl chloride hood at its tip. Many pieces of food were found to be trapped in the esophagus. These were removed using tripod forceps or aspirated into the hood. The internal diameter of the stenotic segment was as small as 1 or 2 mm, and it was difficult to advance the endoscope past the stenosis. The endoscope was withdrawn, and the attached hood was removed and replaced with a transparent cap. This provided clear visualization of the mucosal surface of the stenotic segment, which could not be examined using any conventional device, permitting the stenosis to be relieved.


*Diagnostic and Therapeutic Endoscopy*, Vol. 5, pp. 131–135
Reprints available directly from the publisher
Photocopying permitted by license only

© 1999 OPA (Overseas Publishers Association) N.V.
Published by license under
the Harwood Academic Publishers imprint,
part of The Gordon and Breach Publishing Group.
Printed in Singapore.

### A New Endoscopic Technique for Examination of Esophageal Stenosis: The Funnel-shaped Transparent Cap Technique

O. KATAYAMA[a,*], N. WAKABAYASHI[a], S. ISHIHAMA[b] and I. OHI[c]

[a]*Clinical Laboratory, Saitamaken-Saiseikai Kurihashi Hospital, Kouemon 714-6, Kurihashi-Cho, Kitakatsushika-gun, Saitama 349-1105, Japan;* [b]*Endoscope Technical Division, Toshiba Medical Co., Tokyo 113-8456, Japan;* [c]*Laboratory, Tokyo Women's Medical College Daini Hospital, Tokyo 116-0011, Japan*

*(Received 25 May 1998; In final form 18 June 1998)*

**We have devised a funnel-shaped transparent cap for the endoscopic diagnosis and treatment of stenosis in the digestive tract. This funnel-shaped cap is made of highly transparent methacrylic resin. A 73-year-old woman with reflux esophagitis (categorized as grade D by the Los Angeles Classification) visited our hospital with the chief complaint of dysphagia. She was examined using an endoscope equipped with a transparent vinyl chloride hood at its tip. Many pieces of food were found to be trapped in the esophagus. These were removed using tripod forceps or aspirated into the hood. The internal diameter of the stenotic segment was as small as 1 or 2 mm, and it was difficult to advance the endoscope past the stenosis. The endoscope was withdrawn, and the attached hood was removed and replaced with a transparent cap. This provided clear visualization of the mucosal surface of the stenotic segment, which could not be examined using any conventional device, permitting the stenosis to be relieved.**

*Keywords:* Esophageal stenosis, Funnel-shaped transparent cap, Reflux esophagitis, Transparent hood

### INTRODUCTION

We have developed a prototype funnel-shaped transparent cap for the endoscopic diagnosis and treatment of malignant or benign stenosis in the digestive tract. We used an endoscope equipped with this cap at its tip to examine a patient with severe esophageal stenosis secondary to reflux esophagitis. The mucosa within the stenotic segment, which could not be observed using any conventional device, was clearly visualized, permitting the stenosis to be relieved.

* Corresponding author. Tel.: 0480-52-3611. Fax: 0480-52-1348. E-mail: saisei@mx7.mesh.ne.jp.

### INSTRUMENT AND METHODS

A funnel-shaped transparent cap was devised based on the idea of the Endo-Olive method, developed by Inoue and Takeshita [1]. A highly transparent methacrylic test tube (10 ml) with a heat distortion temperature of 90°C and a funnel-shaped end was cut with a coping saw to obtain the funnel-shaped part. A small hole was made at the tip of the funnel-shaped part using a hand drill. The tip was filed smooth and heated with a gas burner to round the edges (Fig. 1).

An electronic endoscope for the upper digestive tract (TGI-3680D, Toshiba Corporation, Tokyo) incorporating a 410,000-pixel charge-coupled device (CCD) was used. This endoscope has a 120° angle of view, a tip diameter of 10.8 mm, and a shaft diameter of 10.5 mm. The tip can be angulated 210° in the upward direction, 90° in the downward direction, and 100° in both the left and right directions.

The funnel-shaped transparent cap described above was placed over the tip of the endoscope and held in place with surgical tape (Fig. 2).

### CASE REPORT

The case presented here is a 73-year-old woman with reflux esophagitis categorized as grade D by the Los Angeles Classification [2]. This patient had been diagnosed as having esophageal stenosis 8 months previously and underwent balloon dilatation. She visited our hospital on February 3, 1998, with a chief complaint of dysphagia since the morning of that day. She had eaten a breakfast consisting of sliced raw fish and thin pieces of boiled fish paste and afterwards was unable to drink water.

She was examined using an electronic endoscope equipped with a transparent vinyl chloride hood [3] at its tip. Many pieces of food trapped in the esophagus were seen, and they were removed using tripod forceps or aspirated into the hood. Active grade-D reflux esophagitis with a white coating was observed on the oral side of the stenotic segment. The internal diameter of the stenotic segment was as small as 1 or 2 mm, and it was difficult to advance the endoscope past the stenosis (Fig. 3).

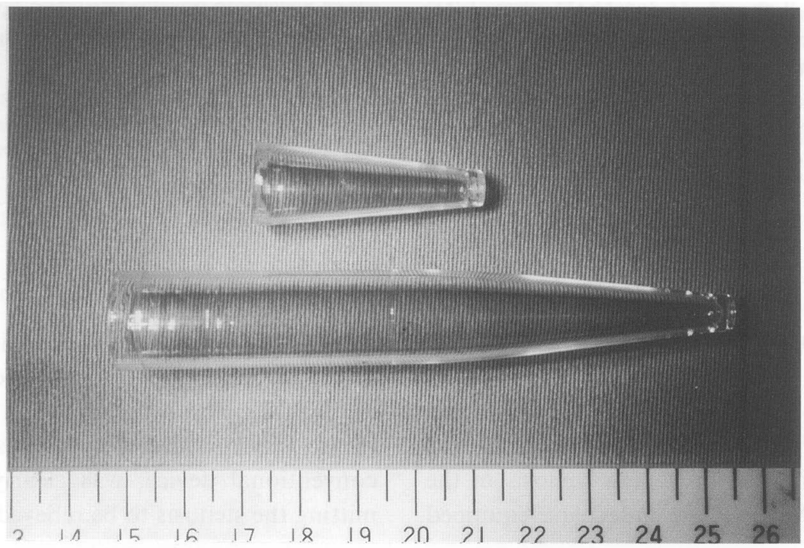

FIGURE 1 A prototype funnel-shaped transparent cap and a methacrylic test tube (10 ml).

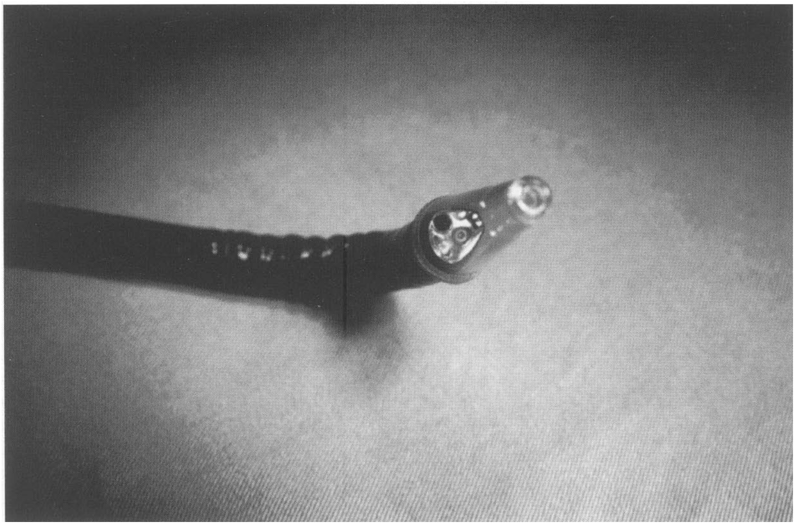

FIGURE 2 The funnel-shaped transparent cap placed over the tip of the endoscope and held in place with surgical tape.

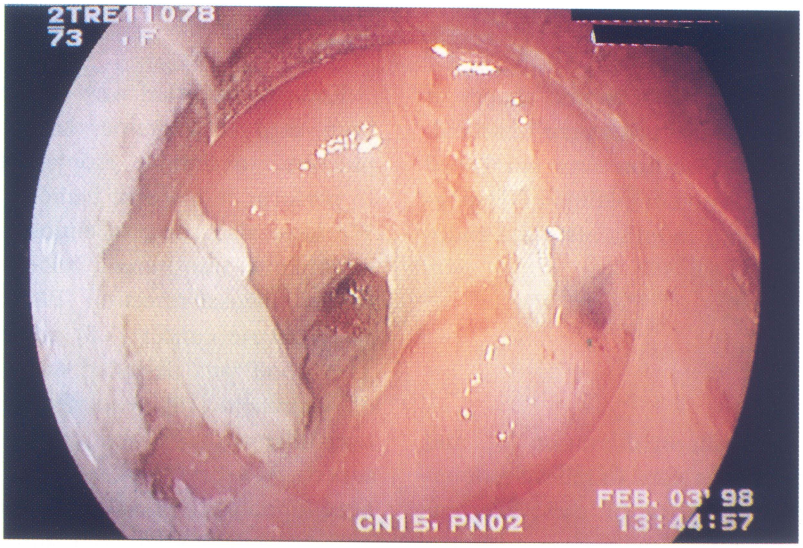

FIGURE 3 An endoscopic picture of the oral side of the stenotic segment using a transparent hood.

The endoscope was withdrawn, and the hood was replaced with the funnel-shaped transparent cap. The endoscope fitted with the funnel-shaped transparent cap passed smoothly through the pharyngoesophageal junction with clear visualization and reached the oral end of the stenotic segment. Then, the endoscope was further advanced against resistance with great care. The mucosa of the stenotic segment was stretched and unfolded over the entire surface of the cap. As the endoscope was carefully advanced, the resistance abruptly dropped and the fornix became visible. The endoscope was pulled back past the stenotic segment and carefully advanced again. The degree

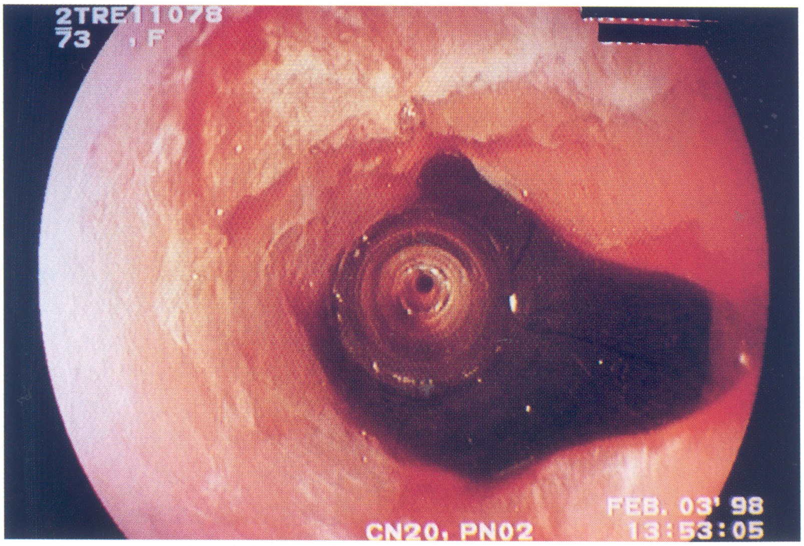

FIGURE 4 An endoscopic picture of mucosal bleeding and tears within the stenotic segment using the funnel-shaped transparent cap.

of mucosal bleeding and the sizes and shapes of tears within the stenotic segment were observed, and the absence of perforation was confirmed (Fig. 4). The patient has no complaints of dysphagia at the present time, 3 months after the endoscopic procedure.

### DISCUSSION

Bougie's method [4], balloon dilatation [5], and stent therapy [6] have been used to relieve esophageal stenosis. However, none of these conventional methods permit visualization of the mucosal surface within the stenotic segment using an endoscope, and these methods often require the use of fluoroscopy. The highly transparent methacrylic cap that we have devised has the advantage of permitting endoscopic visualization of the mucosa within the stenotic segment, as well as in other areas. In addition, since there is a small hole in the tip of the cap, the lens at the tip of the endoscope can be cleaned using the air/water channels of the endoscope.

Endoscopes fitted with a hood at the tip have been used to examine the pharyngoesophageal junction, to remove esophageal foreign bodies, to perform endoscopic hemostasis, and so on [3]. We have also used an endoscope equipped with an oblique transparent hood to perform endoscopic mucosal resection (EMR) in patients with early gastric cancer [7,8] and rectal tumors [9]. This hood was designed based on the concepts of Makuuchi [10], who developed a transparent over-tube with an oblique tip for the EMR of superficial esophageal cancer. The hood has also been found to be useful for the removal of esophageal foreign bodies, but not in patients with severe stenosis in the digestive tract. Since the prototype transparent cap fabricated in the present study has a funnel-shaped tip, rather than a hood, an endoscope fitted with this cap at its tip can be advanced through the stenotic segment like a snow-plow or an ice-breaker as it gently tears the mucosa. Moreover, stenosis in the digestive tract can be safely examined and treated using an endoscope, assuming that the operator exercises sufficient care, because the mucosal tears that are formed can be examined endoscopically.

### *References*

[1] Inoue, H. and Takeshita, K. EMRC method, EVL-LC method, and Endo-Olive method. *Gastroenterol. Endosc.* 1997; **39**: 2062.

[2] Dent, J. and Tytgut, G.N.T. Proposed Los Angeles system for classification of reflux oesophagitis. In a meeting held in conjunction with the 10th World Congress of Gastroenterology, Los Angeles, USA, 5 October, 1994.

[3] Ozaki, M. and Otsuka, S. The utility of a transparent hood in endoscopic surgery for the treatment of stenosis of the upper gastrointestinal tract. *Gastroenterol. Endosc.* 1994; **36**: 1455–1462.

[4] Celestin, L.R. and Campbell, W.B. A new and safe system for esophageal dilatation. *Lancet* 1981; **1**: 74–75.

[5] Shemesh, E. and Czerniak, A. Comparison between Savary-Gilliard and balloon dilatation of benign esophageal strictures. *World J. Surg.* 1990; **14**: 518–522.

[6] Knyrim, K., Wagner, H.J., Bethge, N. *et al.* A controlled trial of an expansile metal stent for palliation of esophageal obstruction due to inoperable cancer. *New Engl. J. Med.* 1993; **329**: 1302–1307.

[7] Katayama, O., Oguri, K., Ohkubo, Y. *et al.* Endoscopic mucosectomy of gastric cancer by use of oblique transparent hood; indication and limitations. *Prog. Dig. Endosc.* 1992; **41**: 154–157, 387.

[8] Katayama, O., Kato, A., Honda, H. *et al.* Endoscopic mucosal resection by the oblique transparent hood technique. *Medical Review* 1996; **62**: 14–19.

[9] Katayama, O. and Honda, H. Endoscopic mucosal resection (EMR) for rectal tumors-oblique transparent hood technique. *Dig. Endosc.* 1997; **9**: 249.

[10] Makuuchi, H. Endoscopic mucosal resection for early esophageal cancer. *Dig. Endosc.* 1996; **8**: 175–179.

